# Editors’ Book Table

**Published:** 1874-04

**Authors:** 


### Editors' Book Table.

NOTE. —All works reviewed in the columns of the CHICAGO MEDICAL JOURNAL may be found in the extensive stock of W. B. KEEN, COOKE & Co. whose catalogue of Medical Books will be sent to any address upon request.]

BOOKS RECEIVED.

*The Puerperal Diseases*—Clinical Lectures delivered at Bellevue Hospital. By FORDYCE BARKER, M.D., Clinical Professor of Midwifery and the Diseases of Women at Bellevue Hospital Medical College, etc., etc. New York: D. Appleton & Co. 1874.

*An Introduction to Physical Measurements*, with Appendices on absolute Electrical Measurement, etc. By Dr. F. KOHLRAUSCH, Professor-in-Ordinary at the Grand Ducal Polytechnic School of Darmstadt. Translated from the second German edition, by Thomas Hutchinson Wallen, B.A., B. Sc., and Henry Richardson Proctor, F.C.S. New York: D. Appleton & Co. 1874.

PAMPHLETS RECEIVED.

*The Relations of Colorado to Pulmonary Consumption.* By THOS. E. MASSEY, A.M. M.D. Denver. 1874.

*The Sixth Annual Report of Toronto Eye and Ear Infirmary.* 1873.

*The Second Biennial Report of the Board of State Commissioners of Public Charities of the State of Illinois.* December, 1872.

*Contributions to the Natural History of Specific Yellow Fever, etc., etc.* By JOSEPH JONES, Professor of Chemistry and Clinical Medicine, University of Louisiana. Reprinted from New Orleans Medical and Surgical Journal—Jan. 1874.

*Catalogue of Jefferson Medical College, Philadelphia.* Session 1873–4.

*Constants of Nature.* Smithsonian Miscellaneous Collections, 255. Part 1. Specific Gravities, Boiling and Melting Points, and Chemical Formulæ.

*Epidemic Diseases as dependent on Meteorological Influences.* By C. SPINZY, M.D., St. Louis.

*Catalogue* of Books and Pamphlets relating to America, etc.—Robert Clarke & Co., Cincinnati.

*Medico-Legal Society, New York*—Inaugural Address of CLARKE BELL, Esq., Nov. 26, 1873, President of the Society.

*Dictionary* of Elevations and Climatic Register of United States. By J. M. TONER, M.D.

*Transactions* of Wisconsin State Medical Society.

*Report of Brigham Hall*, a Hospital for the Insane. Canandaigua, N. Y., 1874.

JOURNALS RECEIVED.

The American Chemist, Philadelphia—January.
" American Medical Journal—January.
" American Journal of Syphilography and Dermatology—Jan.
" Atlanta Medical and Surgical Journal—Feb., March.
" American Practitioner, Louisville—March.
" Am. Naturalist (Salem) Peabody Academy of Science—Mar.
" Boston Medical and Surgical Journal—March, 1874.
" Boston Journal of Chemistry—March.
" Canada Medical and Surgical Journal—February.
" Canada Medical Record—February.
" Central Law Journal—February 19.
" Clinic, Cincinnati—Feb. 14, 21, 28, Mar. 7, 14.
" Cincinnati Lancet and Observer—Feb. and March.
" Detroit Review of Medicine and Pharmacy—March, 1874.
" Dental Cosmos, Philadelphia—Jan., Feb., Mar.
" Druggists' Circular, New York—Mar.
" Eclectic Medical Journal, Cincinnati—Feb. and March.
" Indiana Journal of Medicine—Feb.
" Kansas City Medical Journal—Feb.
" London Lancet—Feb.
" Medical Herald, Leavenworth—Feb.
" Medical Record of New York—Jan. and Feb., Mar. 2.
" Medical Record of Canada (Montreal)—Feb.
" Medical and Surgical Reporter, Philadelphia—Feb. 7, 14, 28, Mar. 7, 14.
" Medical Times, Philadelphia—Feb. 14, 28, March 7, 14.
" Medical Examiner, Chicago—Mar. 1.
" Medical News and Monthly, Philadelphia—March.
" Nashville Journal of Medicine and Surgery—Feb.
" New Orleans Medical and Surgical Journal—Jan.

The North-Western Medical and Surgical Journal—March.
" New York Medical Journal—March.
" Pharmacist, Chicago—March.
" Physician and Pharmacist—March, 1874.
" Practitioner, London—Feb.
" Richmond and Louisville Med. and Surgical Journal—Feb.
" Southern Medical Record, Atlanta—Jan., Feb.
" United States Med. and Surgical Journal, Chicago—Jan.
Le Progres Medicale, Paris—Vol. 2, Nos. 3, 4, 5.

---

